# UAV Onboard STAR-RIS Service Enhancement Mechanism Based on Deep Reinforcement Learning

**DOI:** 10.3390/s25061943

**Published:** 2025-03-20

**Authors:** Junjie Yan, Yichen Xu, Haohao Yuan, Chunhua Xue

**Affiliations:** 1 School of Electronic Engineering, Guangxi University of Science and Technology, Liuzhou 545006, China; yanjj@gxust.edu.cn (J.Y.); qz1144017231@gmail.com (Y.X.); xue@gxust.edu.cn (C.X.); 2 Guangxi Key Laboratory of Multidimensional Information Fusion for Intelligent Vehicles, Liuzhou 545006, China

**Keywords:** STAR-RIS, UAV-enhanced edge services, resource allocation, deep reinforcement learning

## Abstract

UAVs and reconfigurable intelligent surfaces (RISs) have emerged as promising solutions to enhance communication coverage and performance. However, existing studies primarily focus on optimizing the amplitude and phase shift of a STAR-RIS without considering the impact of varying UAV hovering angles on signal reflection and transmission. In this paper, we propose a novel STAR-RIS-assisted UAV service enhancement mechanism that dynamically adjusts reflection/transmission regions based on the real-time user distribution, significantly improving the channel quality for both edge and occluded users. This work is the first to jointly optimize the phase and amplitude of the STAR-RIS, the UAV flight trajectory, and the hovering angle, addressing the critical challenge of co-channel interference caused by dynamically partitioned service areas. The complex optimization problem is decomposed into subproblems, where the UAV flight trajectory is optimized using the Chained Lin–Kernighan (CLK) algorithm and the STAR-RIS parameters and UAV hovering angle are optimized using the TD3 algorithm. The experimental results show that the proposed mechanism effectively reduces the system service time and user transmission time, outperforming traditional methods.

## 1. Introduction

The advent of sixth-generation (6G) communication technology has precipitated the practical implementation of applications such as autonomous driving, telemedicine, and the Internet of Things (IoT), thereby metamorphosing them from theoretical constructs into a tangible reality. These applications demand stringent performance requirements, including ultra-low latency, ultra-high reliability, and a high bandwidth [1]. However, traditional terrestrial communication systems are vulnerable to non-line-of-sight (NLoS) issues in complex environments. This is primarily due to the fixed location of the base station, which results in channel fading and the deterioration of the channel quality. Consequently, it is essential to explore new communication architectures and optimization strategies to mitigate NLoS impacts and improve system reliability and efficiency.

UAVs are seen as a promising solution to overcome communication coverage and capacity limitations in remote and challenging environments. When used as aerial nodes, UAVs offer flexible positioning at optimal locations, increasing the likelihood of line-of-sight (LoS) communication and improving the channel gain [2]. In addition, UAVs can act as communication relays, extending network coverage, increasing the system capacity, and improving communication reliability, flexibility, and robustness [3,4]. Thus, the potential for UAVs to improve system performance in complex communication scenarios is clear. However, UAV communication systems still face several challenges, including energy constraints, dynamic channel conditions, and interference in dense networks [5].

Recently, reconfigurable intelligent surfaces (RISs) have become one of the key enabling technologies for 6G as an effective solution to overcome the above limitations [6]. RISs have been demonstrated to have the capacity to exercise intelligent control over the wireless propagation environment by dynamically adjusting the reflection and transmission characteristics [7]. This dynamic adjustment has been shown to optimize the signal path and reduce multipath interference and channel fading and thus demonstrates great potential in improving the performance of wireless networks [8]. Furthermore, with the flexibility of UAVs and the programmability of RISs, the formation of an aerial RIS (ARIS) to dynamically optimize propagation paths and channel conditions has become one of the current research hotspots [9,10,11].

However, a significant technical challenge in a traditional RIS is that it only has the capacity to support signal reflection, requiring the user to be on the same side as the transmitter to receive the signal, thereby restricting its flexibility and practical deployment in various wireless communication applications. In addressing this limitation, researchers have proposed a STAR-RIS, a concurrent transmission and reflection reconfigurable intelligent surface technology [12]. Specifically, the amplitude adjustment facilitates the determination of the energy distribution of the incident signal, thereby ensuring that a proportion of the energy is reflected and a proportion transmitted, thus optimizing the coverage in different areas. The phase shift adjustment ensures that the signal is effectively superimposed in the target area, thereby increasing the channel gain and reducing interference.

Furthermore, STAR-RIS-assisted UAV systems are highly dynamic and complex. Deep reinforcement learning (DRL), which enables agents to make sequential decisions based on environmental observations and interactions, thereby learning and adapting to dynamic conditions, has become a vital tool for addressing such intricate challenges. Classical DRL algorithms, such as Deep Q-Networks (DQNs) and Deep Deterministic Policy Gradients (DDPGs), have achieved success in UAV path planning and resource allocation. However, DDPGs often suffer from Q value overestimation, leading to suboptimal policies [13]. The Twin Delayed Deep Deterministic Policy Gradient (TD3) addresses this limitation by introducing clipped double-Q learning, delayed policy updates, and target noise regularization, thereby enhancing stability and convergence [14]. Recent studies have also highlighted TD3’s efficacy in communication networks [15].

Despite significant advances in the aforementioned domains, critical research gaps persist. Firstly, while prior studies have optimized STAR-RIS phase and amplitude configurations, they have largely overlooked the impact of UAV hovering angles on signal reflection/transmission partitioning. This oversight impedes the dynamic adaptation of STAR-RIS region boundaries to user density variations. Secondly, existing frameworks typically assume pre-defined user groupings or static service areas, neglecting the need for real-time adaptation to dynamic user distributions. Finally, despite the effects of coupling the UAV trajectory, STAR-RIS parameters, and hovering angles on system performance, their joint optimization remains unexplored.

In this paper, we consider a communication scenario assisted by an aerial STAR-RIS. In this scenario, the aerial STAR-RIS dynamically adjusts the hovering angle according to the user’s geographical location and communication needs to divide up the reflection and transmission areas. The following specific contributions are made by this paper:

- We propose a UAV-mounted STAR-RIS-assisted communication service enhancement mechanism aimed at improving the channel quality for both edge users and occluded users. This mechanism demonstrates its efficacy by enhancing the efficiency and reliability of the communication system through the joint optimization of the STAR-RIS phase and amplitude, as well as the UAV’s flight trajectory and hovering angle. Additionally, the complexity of the optimization problem is addressed by formulating it as the minimization of the UAV’s total service time.
- The joint optimization problem involving a STAR-RIS and UAVs in a complex, dynamic environment includes integer variables and non-convex constraints, making it a mixed-integer nonlinear programming problem that is challenging to solve using traditional methods. To address this complexity, this paper decouples the original optimization problem into two subproblems, total flight time optimization and total transmission time optimization, thereby obtaining a suboptimal solution.
- The total flight time optimization problem employs the Chained Lin–Kernighan (CLK) algorithm to determine a trajectory that minimizes the flight time, following the delineation of the UAV’s service area using the DBSCAN algorithm. For the total transmission time optimization problem, the phase, amplitude, and UAV hovering angle of the STAR-RIS are optimized using the TD3 algorithm to minimize the transmission time.

The remainder of this document is structured as follows: Firstly, in Section 2, we review the work related to this paper. Then Section 3, presents the system model and the joint STAR-RIS phase and amplitude, UAV flight trajectory, and hovering angle problems. In Section 4, the optimization problem-solving method is presented and analyzed. A performance evaluation is presented in Section 5, and conclusions are drawn in Section 6.

## 2. Related Work

In this section, a summary of the relevant work is provided, with a focus on three aspects: UAV-based communication networks, RIS-assisted UAV networks, and STAR-RIS-assisted wireless communication.

### 2.1. UAV-Based Communication Networks

Recent years have seen UAVs being used in various communication scenarios and playing different roles. In [16], the authors proposed a dual-role UAV application model, as an edge server to assist user devices in processing computing tasks, and as a relay to further offload computing tasks to access points. In [17], the application of UAVs in terrestrial sensor networks was explored, achieving the integration of information transmission and charging functions. In [18], a DQN algorithm based on deep reinforcement learning was proposed for UAV-assisted IoT data collection systems, which effectively reduced the system packet loss rate through the online optimization of UAV flight control and data scheduling strategies. In [19], the application prospects of UAV communications in the millimeter wave band are systematically outlined, with performance evaluation metrics and analysis methods elaborated and key technologies and potential solutions proposed for different application scenarios. In addressing the need for effective disaster communications, a UAV deployment scheme was proposed in [20] to ensure the maximum coverage of ground nodes. However, none of these studies fully considered the impact of RISs on the wireless channel.

### 2.2. RIS-Assisted UAV Networks

The utilization of RISs in UAV communication networks has been the subject of extensive research in the literature [21,22,23,24,25,26]. Research has focused on multiple metrics, including the enhancement of data transmission rates, the improvement of communication reliability, and the augmentation of communication security. In [21], a classic RIS-assisted UAV communication framework was explored, aiming to maximize the average achievable rate. This optimization was achieved by jointly adjusting the UAV trajectory and the RIS passive beamforming design. In [22], a multi-UAV combined RIS-assisted mobile edge computing (MEC) system was addressed. Using a multi-agent deep reinforcement learning framework, the study focused on minimizing system latency and ensuring fairness among user devices by optimizing the computing offloading strategy and UAV trajectory. In [23], secure computation in a UAV-RIS-assisted multi-layer MEC system was addressed, and a full-duplex active eavesdropper was introduced. In [24], UAV-assisted communication with 3D trajectories was investigated, and a D3QN-based optimization algorithm was proposed to jointly optimize bandwidth allocation, RIS phase shifts, and 3D coordinates to maximize energy efficiency. In [25], a cooperative ARIS-assisted MEC system with two UAVs was considered. To enhance the system energy efficiency, a DDQN algorithm was used to optimize UAV trajectories, ARIS phase shifts, computation offloading strategies, and resource allocation. In [26], the authors presented a multi-antenna covert communication system enhanced by UAV-RIS, with which they creatively optimized beamforming, RIS phase shifts, and the UAV trajectory to maximize the worst-case covert transmission rate, even under imperfect CSI conditions.

### 2.3. STAR-RIS-Assisted Wireless Networks

To address the limitation of traditional RISs, where users and base stations must be located on the same side, increasing attention is being directed towards the STAR-RIS. The resource allocation schemes for STAR-RIS-aided multi-carrier systems in both orthogonal multiple access (OMA) and non-orthogonal multiple access (NOMA) scenarios are discussed in [27]. In [28], an optimization scheme for vehicular communication systems based on the STAR-RIS is proposed to improve data rates through the joint optimization of factors such as spectrum allocation and the amplitude and phase shift of STAR-RIS elements. However, the strong coupling characteristics of non-ideal CSI and non-ideal serial interference cancellation in practical communication scenarios make it impossible to accurately measure the safety performance of STAR-RIS-assisted NOMA transmission systems. To address this challenge, a fusion algorithm is proposed in [29] to maximize the system’s minimum safe transmission rate. In [30], the integration of the STAR-RIS with UAV NOMA communication networks was explored. This work aimed to maximize the user throughput by jointly optimizing UAV trajectories, the passive beamforming of the STAR-RIS, and time and power allocation, addressing the practical needs of disaster emergency communications. In [31], an MEC service enhancement scheme deploying the STAR-RIS on a UAV as a mobile relay is proposed. This approach combines the rapid deployment and mobility of UAVs with the advantages of the STAR-RIS to enhance spectral efficiency, expand coverage, and reduce the system’s energy consumption. A collaborative optimization scheme for emergency communication networks integrating the STAR-RIS and UAVs is also proposed in [32]. Experimental results demonstrate that this approach significantly enhances the average video streaming capability of users in disaster scenarios.

However, the majority of existing studies on STAR-RIS-aided UAV networks assume that the UAV service area is decentralized and that the UAV needs only to design an optimal flight path for the discrete service area. Moreover, the existing literature assumes that reflective and transmissive users are pre-determined, focusing optimization on the amplitude and phase shift of the STAR-RIS. However, these studies do not consider the impact of varying UAV hovering angles on STAR-RIS signal reflection and transmission. When the UAV hovering angle changes, the reflection and transmission service area of the STAR-RIS adjusts dynamically. This adjustment increases the number of users in the reflecting or transmitting area, which can cause significant co-channel interference and degrade system performance. Therefore, further exploration is required into the division of reflection and transmission regions when deploying the STAR-RIS on UAVs.

## 3. System Model and Problem Formulation

**Figure 1:**
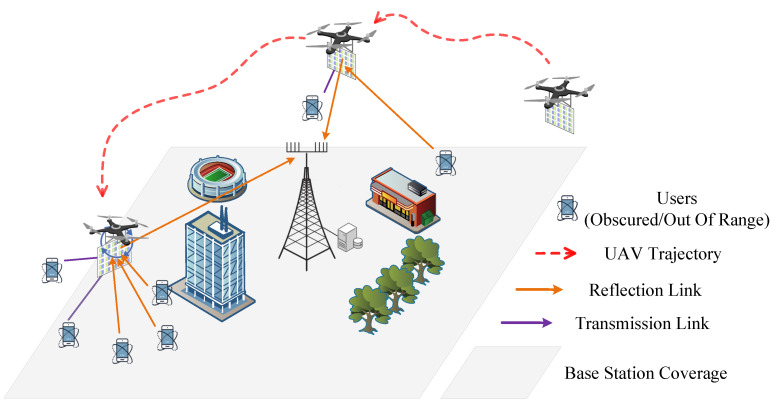
STAR-RIS-assisted UAV edge service enhancement system.

As shown in Figure 1, this paper considers a UAV service enhancement system supported by a STAR-RIS. The system consists of several user devices, k,k∈K={1,…,K}, and a UAV equipped with a STAR-RIS and a ground base station. The coordinates of the base station BS and the user are denoted by $Z_{b}=\left[x_{b},y_{b},H_{b}\right]$ and $Z_{k}=\left[x_{k},y_{k},0\right]$ respectively. The UAV *u* provides services to the users at different time intervals within a time period, *T*. For analysis purposes, the UAV flight period is divided into *N* equidistant time intervals, i.e., N={1,...,n,...,N}, with a step size of Δt, which is the time period T=NΔt. The STAR-RIS, consisting of *M* reflection/transmission units, can establish a good channel between the user and the base station. The phase of each reflection/transmission unit of the STAR-RIS can be controlled by the UAV, so the phase shift matrix of the STAR-RIS for reflection and transmission in the *n* time slot can be expressed as follows [12]:

(1) $$\Theta_{rfl}\left[n\right]=\mathrm{diag}\left(\sqrt{\beta_{rfl}^{1}\left[n\right]}e^{j\theta_{rfl}^{1}\left[n\right]},...,\sqrt{\beta_{rfl}^{M}\left[n\right]}e^{j\theta_{rfl}^{M}\left[n\right]}\right)$$

(2) $$\Theta_{rfr}\left[n\right]=\mathrm{diag}\left(\sqrt{\beta_{rfr}^{1}\left[n\right]}e^{j\theta_{rfr}^{1}\left[n\right]},...,\sqrt{\beta_{rfr}^{M}\left[n\right]}e^{j\theta_{rfr}^{M}\left[n\right]}\right)$$

where $\beta_{rfl}^{m}\left[n\right],\beta_{rfr}^{m}\left[n\right]\in \left[0,1\right],m\in M=\left\{1,\ldots ,M\right\}$ represents the amplitude of the *m* element reflection coefficient and transmission coefficient in the time slot *n*, and $\beta_{rfl}^{m}\left[n\right]+\beta_{rfr}^{m}\left[n\right]=1,\theta_{rfl}^{m}\left[n\right],\theta_{rfr}^{m}\left[n\right]\in \left[0,2\pi \right)$ represents the phase shift value of the *m* element reflection and transmission.

### 3.1. Mobile Model

Without the loss of generality, all communication nodes are placed in a three-dimensional Cartesian coordinate system. To ensure safety and maintain reliable LoS connectivity between the UAV, BS, and users [33], it is assumed that the UAV provides services at a fixed altitude, $H_{u}$, and based on the systems proposed in [25,26,31,32], the UAV and STAR-RIS are treated as a rigidly connected entity, ensuring their horizontal coordinates and altitude remain identical. In the time slot *n*, the horizontal coordinates of the UAV are denoted as $Z_{u}\left[n\right]=\left[x_{u}^{n},y_{u}^{n}\right]$, which satisfies $x_{u}^{n}\in \left[0,x_{u}^{max}\right],y_{u}^{n}\in \left[0,y_{u}^{max}\right]$. Furthermore, following the shortest path planned by the CLK algorithm (Equations (19)–(21)), the UAV flies at a constant speed of $v_{u}$ between hover points, ensuring the minimization of the total flight time.

**Figure 2:**
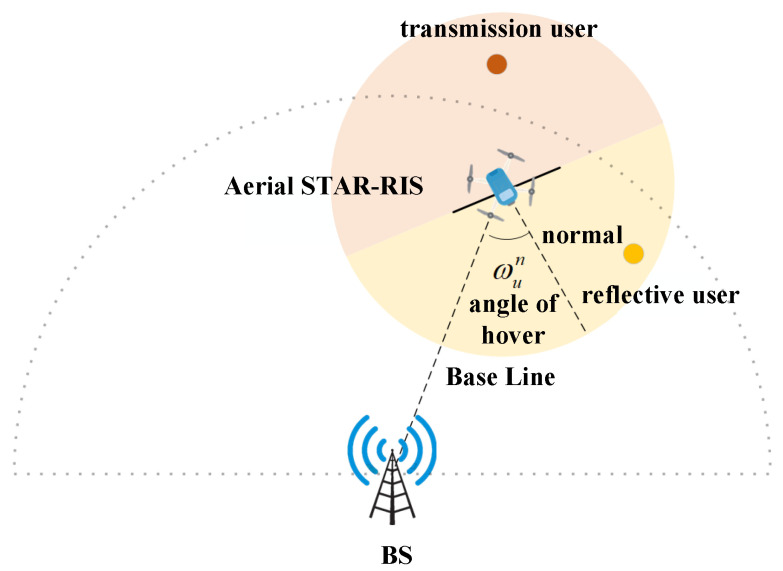
Relationship between UAV hovering angle and reflective transmission service area.

As illustrated in Figure 2, the STAR-RIS is vertically deployed beneath the UAV with its reflective surface oriented along the UAV’s heading direction, while the transmissive surface faces the opposite direction. It is assumed that the UAV can adjust the hover angle $\omega_{u}^{n}$ in the horizontal direction while hovering. The hovering angle affects the division of the STAR-RIS reflection/transmission service area, which in turn affects the change in the number of users within the reflection or transmission area. As the number of users in the echo or transmission area increases, the co-frequency interference between multiple users will increase significantly, resulting in a decrease in system performance. Therefore, optimizing $\omega_{u}^{n}$ is of paramount importance. In this paper, the angle of the surface normal vector of the STAR-RIS pointing to the direction of the base station when the UAV is hovering is taken as the benchmark angle. At this time, the surface normal vector can be expressed as

(3) $$\upsilon_{0}=\frac{\left(x_{b}-x_{u}^{n},y_{b}-y_{u}^{n}\right)}{\sqrt{{\left(x_{b}-x_{u}^{n}\right)}^{2}+{\left(y_{b}-y_{u}^{n}\right)}^{2}}}$$

When the UAV changes the hovering angle, the rotated normal vector can be obtained by applying the rotation matrix in the 2D plane to the reference normal vector:

(4) $$\upsilon =\left[\begin{array}{cc} \cos\omega_{u}^{n} & -\sin\omega_{u}^{n}\\ \sin\omega_{u}^{n} & \cos\omega_{u}^{n} \end{array}\right]\cdot \upsilon_{0}$$

The adjustment range of the hovering angle is defined as follows: $\left(-\frac{\pi }{2},\frac{\pi }{2}\right)$.

### 3.2. Service User Model

In the proposed system, UAVS are required to assist the BS in serving users with transmission requirements. Specifically, UAVS assist users who are unable to establish an effective link with the base station. This includes users outside the base station’s coverage area or users within the base station’s coverage area who experience link quality insufficient to support reliable communication, denoted by $C_{k,u}$. The following equation specifically expresses this:

(5) $$C_{k,u}=\left\{\begin{array}{cc} 1, & \mathrm{others},\\ 0, & ∥Z_{b}-Z_{k}{∥}_{2}<\rho_{b}\;\mathrm{and}\;off_{k,b}=0 \end{array}\right.$$

where $off_{k,b}$ indicates whether an effective link can be established between the user with a transmission requirement and the base station. A value of $off_{k,b}=1$ indicates the establishment of an effective link between the user with a transmission requirement and the base station; conversely, a value of $off_{k,b}=0$ indicates the opposite.

### 3.3. Communication Model

As previously stated, the primary focus of this paper is the UAV service enhancement system assisted by a STAR-RIS. Consequently, the construction of two links is given primary consideration in the proposed system: one is the link transmitting users to the aerial STAR-RIS (ASTAR-RIS), and the other is the link from the ASTAR-RIS to the BS. It is assumed that only LoS channels exist between the user who transmitted in the first time slot via a UAV and the BS, and thus, it is assumed that the channel fading experienced here only involves the LoS component Rice fading. Consequently, the wireless channel gain $G_{r,b}\left[n\right]$ from the ASTAR-RIS to the BS in the time slot *n* can be expressed as

(6) $$G_{r,b}\left[n\right]=\sqrt{\varphi d_{r,b}^{-\xi_{r,b}}\left[n\right]}\sqrt{\frac{\Upsilon }{1+\Upsilon }}g_{r,b}^{\mathrm{LoS}}\left[n\right]\in C^{M\times 1_{\leftarrow }}$$

where $\xi_{r,b}$ represents the path loss index from the ASTAR-RIS to the base station, represents the distance from the ASTAR-RIS to the BS, and $d_{r,b}\left[n\right]=\sqrt{{\left(x_{u}^{n}-x_{b}\right)}^{2}+\left(y_{u}^{n}-y_{b}\right)+{\left(H_{u}-H_{b}\right)}^{2}}$ represents the deterministic LoS component between the ASTAR-RIS and BS at time $g_{r,b}^{\mathrm{LoS}}\left[n\right]$. It can be expressed as [25]

(7) $$g_{r,b}^{\mathrm{LoS}}\left[n\right]={\left[1,e^{-j\frac{2\pi }{\nu }\hat{d}\cos\phi_{r,b}^{n}},\ldots ,e^{-j\frac{2\pi }{\nu }\hat{d}\left(M-1\right)\cos\phi_{r,b}^{n}}\right]}^{T}$$

where ν represents the carrier wavelength, $\hat{d}$ represents the spacing between elements, and $\cos\phi_{r,b}^{n}=\frac{\left(x_{u}^{n}-x_{b}\right)\cdot \upsilon_{x}+\left(y_{u}^{n}-y_{b}\right)\cdot \upsilon_{y}}{d_{r,b}\left[n\right]}$ represents the cosine of the signal’s exit angle.

Due to the characteristics of the STAR-RIS, the user-to-ASTAR-RIS link is divided into two parts. For the time slot *n*, the wireless channel gain of the reflection/transmission path transmitting user $G_{k,r}\left[n\right]$ to the ASTAR-RIS can be expressed as

(8) $$G_{k,r}\left[n\right]=\sqrt{\varphi d_{k,r}^{-\xi_{k,r}}\left[n\right]}\sqrt{\frac{\Upsilon }{1+\Upsilon }}g_{k,r}^{\mathrm{LoS}}\left[n\right]\in C^{1\times M}$$

where $\xi_{k,r}$ represents the path loss index from the user to the ASTAR-RIS, and $d_{k,r}\left[n\right]$ represents the distance from the user to the ASTAR-RIS, and $g_{k,r}^{\mathrm{LoS}}\left[n\right]$, which is the deterministic LoS component between the user *k* and the ASTAR-RIS, is expressed as

(9) $$g_{k,r}^{\mathrm{LoS}}\left[n\right]={\left[1,e^{-j\frac{2\pi }{\lambda }\hat{d}\cos\phi_{k,r}^{n}},\ldots ,e^{-j\frac{2\pi }{\lambda }\hat{d}\left(M-1\right)\cos\phi_{k,r}^{n}}\right]}^{T}$$

where $\cos\phi_{k^{\prime },r}^{n}=\frac{\left(x_{k}-x_{u}^{n}\right)\cdot \upsilon_{x}+\left(y_{k}-y_{u}^{n}\right)\cdot \upsilon_{y}}{d_{k,r}\left[n\right]}\cdot$ is the cosine of the angle of incidence of the signal. Therefore, the channel gain from the user to the BS in the reflected path can be expressed as

(10) $$G_{k,b}^{rfl}\left[n\right]=G_{k,r}{\left[n\right]}^{T}\Theta_{rfl}\left[n\right]G_{r,b}\left[n\right]$$

Similarly, the channel gain in the transmission path from the user to the BS can be expressed as

(11) $$G_{k,b}^{rfr}\left[n\right]=G_{k,r}{\left[n\right]}^{T}\Theta_{rfr}\left[n\right]G_{r,b}\left[n\right]$$

In summary, the reachable user-to-BS transmission rate can be expressed as

(12) $$R_{k,b}^{\delta }\left[n\right]=B_{k,b}\log_{2}\left(1+\frac{P_{k}|G_{k,b}^{\delta }\left[n\right]{|}^{2}}{\sum_{j=1,j\neq k}^{\hat{j}\delta }P_{q}|G_{q,b}^{\delta }\left[n\right]{|}^{2}+\sigma^{2}}\right),\delta \in \left\{rfl,rfr\right\}$$

where $B_{k,b}$ represents the bandwidth of the subcarrier assigned to the user by the base station.

### 3.4. Time Model

As previously stated, in this scenario, the UAV is required to assist the base station in serving users with transmission needs and formulate corresponding trajectories. Consequently, the total service time of the UAV is divided into two components: the flight time of the UAV and the time spent serving users with transmission needs.

Assuming that the flight path length of the UAV in the time slot *n* is $L_{u}\left[n\right]$, the total flight time of the UAV can be expressed as

(13) $$D_{fly}\left[n\right]=\frac{L_{u}\left[n\right]}{v_{u}}$$

In instances where an obstruction hinders communication or the user’s location lies beyond the base station’s coverage radius, the quality of the wireless connection will be inadequate to satisfy the user’s demand for data transmission. Consequently, the data must be uploaded to the base station via the reflective or transmissive link of the STAR-RIS. This paper proposes the variable $\gamma_{k}\left[n\right]$, which serves to differentiate between users employing reflective and transmissive channels. Specifically, when $\gamma_{k}\left[n\right]=1$, the user establishes a connection with the base station via the reflective link; when $\gamma_{k}\left[n\right]=0$, the user establishes a connection via the transmissive link.

Assuming that the size of the task that the user *k* needs to upload is $S_{k}$, the transmission time $D_{k,b}\left[n\right]$ can be expressed as

(14) $$D_{k,b}\left[n\right]=\gamma_{k}\left[n\right]\frac{S_{k}}{R_{k,b}^{rfl}\left[n\right]}+\left(1-\gamma_{k}\left[n\right]\right)\frac{S_{k}}{R_{k,b}^{rfr}\left[n\right]}$$

In summary, within the designated time slot *n*, the total transmission time of the user is the maximum of the transmission times of each user, which can be expressed as

(15) $$D_{trans}\left[n\right]=\max_{k=\{1,\ldots ,K\}}C_{k,u}D_{k,b}\left[n\right]$$

### 3.5. Problem Formulation

In this section, we propose a methodology for addressing the user’s elevated expectations concerning the quality of links. To this end, we integrate the STAR-RIS phase $\Theta =\{\Theta_{rfl}\left[n\right],\Theta_{rfr}\left[n\right],n\in N\}$, amplitude $\beta =\{\beta_{rfl}\left[n\right],\beta_{rfr}\left[n\right],n\in N\}$, UAV trajectory $Z=\{Z_{u}\left[n\right],n\in N\}$, hovering angle $\omega =\{\omega_{u}^{n},n\in N\}$, and user assignment decision $\gamma =\{\gamma_{k}\left[n\right],n\in N\}$. We then formulate an optimization algorithm aimed at minimizing the total UAV service time. The optimization problem can be modeled as follows:

$$P:\min_{\begin{array}{c} \theta ,\beta ,Z,\omega ,\gamma \end{array}}\;\sum_{n=1}^{N}\left(D_{\mathrm{trans}}\left[n\right]+D_{\mathrm{fly}}\left[n\right]\right)$$

(16) $$\begin{array}{cc} s.t.\;C1:\; & ∥\frac{Z_{u}\left[n+1\right]-Z_{u}\left[n\right]}{\Delta t}∥=v_{u},\;\forall n\in N\\ C2:\; & 0\leq x_{u}^{n}\leq x_{u}^{\max},\;\forall n\\ C3:\; & 0\leq y_{u}^{n}\leq y_{u}^{\max},\;\forall n\\ C4:\; & \gamma_{k}\left[n\right]\in \left\{0,1\right\},\;\forall k,n\\ C5:\; & \beta_{r_{\mathrm{rf}}}^{m}\left[n\right]+\beta_{r_{\mathrm{rf}}}^{m}\left[n\right]=1,\;\forall m,n\\ C6:\; & \theta_{r_{\mathrm{rf}}}^{m}\left[n\right],\theta_{r_{\mathrm{rf}}}^{m}\left[n\right]\in \left[0,2\pi \right),\;\forall m,n\\ C7:\; & \omega_{u}^{n}\in \left(-\frac{\pi }{2},\frac{\pi }{2}\right),\;\forall n \end{array}$$

where C1 signifies the maximum allowable velocity for the UAV, while C2 and C3 delineate the boundaries of the UAV’s operational range. These constraints guarantee that the UAV operates within the prescribed velocity and physical limits while ensuring safety. C4 constitutes a binary constraint that allocates decision variables to users, thereby ensuring that each transmission user utilizes only a single channel per time slot. Constraints C5 and C6 pertain to the reflection and transmission coefficients and phase shift angles of the STAR-RIS, stipulating that the sum of the reflection and transmission coefficients of each element is equivalent to 1 and that the phase shift angle falls within the permissible range. Constraint C7 restricts the UAV hovering angle. However, Problem P is a mixed-integer nonlinear programming (MINLP) problem due to the involvement of high-dimensional mixed variables and non-convex constraints, posing significant challenges for direct optimization. First, the coupling between discrete trajectory variables and continuous RIS parameters creates a highly non-convex solution space. Second, the simultaneous optimization of all the variables demands prohibitive computational resources.

**Figure 3:**
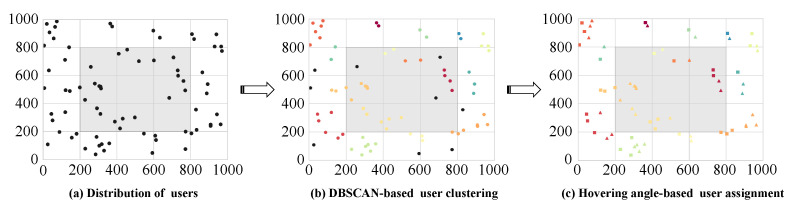
The total optimization process.

Therefore, a strategy was adopted that involved decomposing the original problem into multiple subproblems, each of which was solved separately. The first subproblem encompasses UAV service area division and UAV trajectory planning. As illustrated in Figure 3, we initially determined the distribution of the service area using the DBSCAN algorithm and subsequently determined the optimal UAV trajectory using the algorithm. The subsequent subproblem pertains to the optimization of the amplitude and phase of the STAR-RIS and the hovering angle of the UAV. This subproblem is addressed by employing the TD3 algorithm.

## 4. Proposed Optimization Algorithm

In this section, we provide a comprehensive exposition of the algorithm proposed to address the two aforementioned subproblems.

### 4.1. UAV Time of Flight Optimization Algorithm

The objective of subproblem P1 is to minimize the flight time of the UAV. Therefore, P1 can be expressed as

$$P1:\min_{\begin{array}{c} Z \end{array}}\;\sum_{n=1}^{N}\left(D_{\mathrm{fly}}\left[n\right]\right)$$

(17) $$\begin{array}{cc} s.t.\;C1:\; & ∥\frac{Z_{u}\left[n+1\right]-Z_{u}\left[n\right]}{\Delta t}∥=v_{u},\;\forall n\in N\\ C2:\; & 0\leq x_{u}^{n}\leq x_{u}^{\max},\;\forall n\\ C3:\; & 0\leq y_{u}^{n}\leq y_{u}^{\max},\;\forall n \end{array}$$

Given that the trajectory of the UAV is a dynamically changing process and the coverage area of the UAV is limited, subproblem P1 as a whole is an NP-hard problem, and it is challenging to obtain a solution in polynomial time. To address this challenge, this paper proposes a two-pronged approach. Firstly, it employs the DBSCAN method to segment the non-uniformly distributed users into multiple relatively independent sets. Secondly, it employs the CLK algorithm to optimize the UAV trajectory, thereby identifying the shortest path for the UAV to serve all user sets.

#### 4.1.1. DBSCAN-Based Service Area Classification

DBSCAN is a density-based clustering algorithm that functions independently of a pre-specified number of clusters and is effective in delineating clusters of users from independent users. The algorithm defines the neighborhood of a point by two main parameters, the minimum number of samples, MinPts, and the radius ε, and divides the dataset according to the density of the points [34].

The algorithm process is as follows.

Initially, the parameters MinPts and ε of DBSCAN are established in accordance with the specified scenario. In this paper, ε is delineated as the radius of the service range of the UAV, and MinPts is configured to 2 to guarantee that a minimum of two users are incorporated into the neighborhood of the core point. Subsequently, the density of each transmitting user in the scenario is calculated within the specified range ε. If this density is greater than or equal to MinPts, the user is designated as a core point. In essence, the process commences with the identification of the core point. Thereafter, all users within the designated neighborhood are designated as belonging to the same class. The clustering process is then extended until further extension is no longer feasible. Isolated users, i.e., noisy points, are to be treated separately.

Pursuant to the aforementioned steps, it is possible to divide the transmission users into a number of relatively independent sets. Each set can be represented by $\Omega^{o}$, o∈𝒪={1,…,O}, and the users in each set, $\left\{\Omega_{1}^{o},\Omega_{2}^{o},\ldots ,\Omega_{\hat{k}}^{o}\right\}$, have similar geographic locations and network environments. In this context, denotes the number of users within the current set.

#### 4.1.2. CLK-Based UAV Path Optimization

According to the aforementioned division of the service area of the UAV, a series of hovering points can be obtained. In the strategy of this paper, it is necessary to plan the flight trajectory of the UAV in order to determine the shortest path without violating the constraints, so that the UAV can visit all the hovering points and reach the end point in the shortest time. This problem can be transformed into a no-return traveling salesman problem (NTSP), which is solved in this paper by using the CLK algorithm. The CLK algorithm is a path optimization algorithm that has been enhanced to combine a chain restart mechanism with a local search strategy. This combination of techniques has been shown to be advantageous in the context of solving the large-scale TSP and its variants [35]. The CLK algorithm was selected due to its ability to produce solutions of a quality that is almost optimal, in addition to its capacity to converge more rapidly than stochastic algorithms (e.g., the ant colony algorithm) [36].

It is established that the initial and terminal points of a UAV are designated as $Z_{start}$ and $Z_{goal}$, respectively, with the hovering point designated as $\left\{Z_{u}^{1},Z_{u}^{2},\ldots ,Z_{u}^{o}\right\}$. It is noteworthy that each hovering point, $Z_{u}^{o}$, along with $Z_{start}$ and $Z_{goal}$, is regarded as a node. The hovering point of the UAV corresponds to the center of the current colony, which can be denoted as

(18) $$Z_{u}^{o}=\frac{1}{|\Omega^{o}|}\sum_{k\in \Omega^{o}}Z_{k}$$

The edge $e_{o,o^{\prime }}$ between two nodes represents the path from node $Z_{u}^{o}$ to node $Z_{u}^{o^{\prime }}$. The weight of the edge $d(e_{o,o^{\prime }})$ is the Euclidean distance between the two nodes, which can be expressed as

(19) $$d\left(e_{o,o^{\prime }}\right)=\left|\right|Z_{u}^{o}-Z_{u}^{o^{\prime }}\left|\right|$$

In contrast to the TSP, the NTSP does not necessitate a return to the initial point. Consequently, the introduction of a virtual node, devoid of specific coordinates, becomes imperative. The cost of traversing from this virtual node to the other nodes is either negligible or a substantial value [37]. This guarantees that in the ultimate solution, the virtual node manifests solely as the starting or ending point of the path. The node transfer cost matrix for the NTSP problem can thus be obtained as shown in Table 1:

The subsequent step involves the formulation of a specific solution strategy. Initially, an arbitrary path is generated. Within the LK algorithm, the switching operation corresponds to the operation, that is to say, the selection of edges from the current path to be exchanged to form a new path. Assuming the current path is $\pi =(Z_{virtual},Z_{start},Z_{u}^{1},\ldots ,Z_{u}^{o},Z_{goal},Z_{virtual})$, the switching operation $\chi_{\pi \to \pi^{\prime }}^{\varpi }$ can be defined as the selection of ϖ pairs of nodes and the reconnection of these nodes to form a new path, $\pi^{\prime }$. To quantify the efficacy of the switching operation, it is essential to calculate the benefit of switching, defined as the change in the path length induced by the switching operation. Specifically, the total length of the path π is the sum of the weights of the edges between the nodes, which can be expressed as follows:

(20) $$L\left(\pi \right)=\sum_{(Z_{u}^{o},Z_{u}^{o^{\prime }})\in P}d\left(e_{o,o^{\prime }}\right)$$

The switching gain, therefore, is defined as the difference in length between the new path and the original path after the switching operation.

(21) $$U_{\pi \to \pi^{\prime }}^{\varpi }=L\left(\pi \right)-L\left(\pi^{\prime }\right)$$

A positive value indicates that the path following the switching operation is preferable to the original path, and the algorithm retains the new path $\pi^{\prime }$. Conversely, a negative value signifies that the original path remains unchanged.

The CLK algorithm is a refinement of the LK algorithm that incorporates a restart chain mechanism. A critical aspect of the CLK algorithm involves path perturbation, which aims to disrupt the local optimal structure of the current path by employing perturbation strategies. This process generates new initial solutions, facilitating the continuation of the LK algorithm’s optimization process. This perturbation process typically utilizes random ϖ-opt operations to rearrange the paths. The CLK algorithm generates a series of locally optimal solutions through the successive application of the LK algorithm and the perturbation operations. These solutions are linked to a chain by perturbation and optimization until the path remains static, at which point the final UAV flight trajectory is obtained. The complete algorithm flow is shown in Algorithm 1.
**Algorithm** **1** UAV time of flight optimization algorithm 1:**Input:** ${Ƶ}_{k},\varepsilon ,minPts,{Ƶ}_{\mathrm{start}},{Ƶ}_{\mathrm{goal}}$ 2:Mark all users as unvisited. 3:**repeat** 4:     For each user, calculate all neighboring users within the neighborhood. 5:     **if** neighborhood user ≥minPts **then** 6:        Mark as a core point. 7:        Group users adjacent to the core point into the same cluster. 8:**until** there are no unvisited objects. 9:Obtain user clusters $\Omega^{o}$.10:Calculate hover points using formula (18), and calculate edge weights using formula (19).11:Randomly generate an initial path π, and taking it as the current optimal path $\pi^{*}$.12:For iter = 1 to max iter do13:     Apply the LK algorithm to optimize the current path $\pi^{*}$ and obtain the local optimal path $\pi^{*}$.14:     **if** $U_{\pi }>0$ **then**15:        Update the path $\pi \leftarrow \pi^{*}$.16:     **end if**17:     Perturb the current solution $\pi^{*}\leftarrow \mathrm{new}\;\mathrm{initial}\;\mathrm{solution}$.18:**end for**19:**Output:** Ƶ

### 4.2. Time of Transmission Optimization Algorithm

Subproblem P2 aims to optimize user transmission times by calibrating the phases and amplitudes of the STAR-RIS, the UAV’s hovering angle, and the user assignment strategy, all while maximizing the UAV’s trajectory efficiency. Thus, the P2 problem can be formally stated as

$$P2:\min_{\begin{array}{c} \theta ,\beta ,Z,\omega ,\gamma \end{array}}\;\sum_{n=1}^{N}\left(D_{\mathrm{trans}}\left[n\right]+D_{\mathrm{fly}}\left[n\right]\right)$$

(22) $$\begin{array}{cc} s.t.\;C1:\; & \gamma_{k}\left[n\right]\in \left\{0,1\right\},\;\forall k,n\\ C2:\; & \beta_{r_{\mathrm{rf}}}^{m}\left[n\right]+\beta_{r_{\mathrm{rf}}}^{m}\left[n\right]=1,\;\forall m,n\\ C3:\; & \theta_{r_{\mathrm{rf}}}^{m}\left[n\right],\theta_{r_{\mathrm{rf}}}^{m}\left[n\right]\in \left[0,2\pi \right),\;\forall m,n\\ C4:\; & \omega_{u}^{n}\in \left(-\frac{\pi }{2},\frac{\pi }{2}\right),\;\forall n \end{array}$$

**Figure 4:**
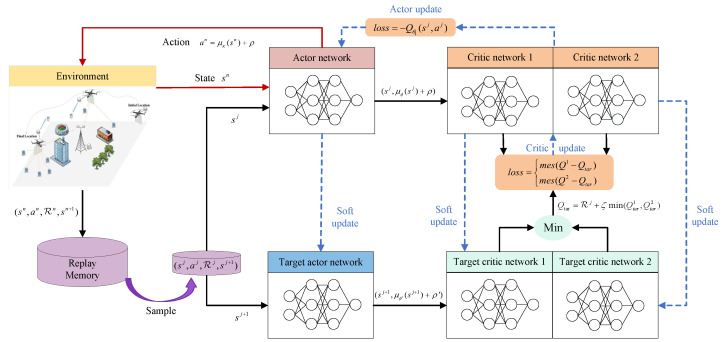
Optimization process based on TD3 algorithm.

However, the phase and amplitude configurations of the STAR-RIS, as well as the hovering angle of the UAV, are subject to dynamic and continuous change. Consequently, due to the non-convexity and high-dimensional complexity of the problem, obtaining its direct solution using conventional methods is usually infeasible. To address this challenge, the TD3 algorithm is employed in this paper as a solution. As illustrated in Figure 4, the TD3 algorithm is a deep reinforcement learning method specifically designed for solving problems in continuous action space, and it has become a popular choice for solving high-dimensional nonlinear optimization problems due to its excellent performance [38].

The TD3 algorithm is generally solved based on the Markov Decision Process (MDP).The MDP can be defined as a quaternion, <S,A,R,P>, which contains the state space, the action space, the state transfer probability, and the reward. In each time slot, *n*, the intelligent body acquires the current state $s^{n}$ from the environment, executes an action, $a^{n}$, interacts with the environment, and consequently arrives at the next state $s^{n+1}$ and receives a reward, $r^{n}$. The following are the specific designs for the state space, action space, and rewards for the intelligent bodies:

- State Space: The set of the agent’s states is denoted by *S*, the agent’s state in the time slot is represented by $s^{n}$, and $s^{n}\in S$ is composed of the current UAV’s coordinates, the STAR-RIS phase and amplitude, and the assignment decision, which is defined as
  (23) $$s^{n}=\left\{Z_{u}\left[n\right],\Theta_{rfl}\left[n\right],\Theta_{rfr}\left[n\right],{\mathrm{fi}}_{rfl}\left[n\right],{\mathrm{fi}}_{rfr}\left[n\right],\omega_{u}^{n}\right\}$$
- Action Space: The set of actions of the agent is denoted by *A*, the agent’s action in the time slot *n* is indicated by $a^{n}$, and $a^{n}\in A$ includes the amplitude factor $\left\{\beta_{rfl}^{m}\left[n\right],\beta_{rfr}^{m}\left[n\right]\right\}$ and phase shift factor $\left\{\theta_{r}^{m}\left[n\right],\theta_{t}^{m}\left[n\right]\right\}$ of the STAR-RIS, as well as the hovering angle $\left\{\omega_{u}^{n}\right\}$ of the UAV. These can all be defined as increments of the current value, with the specific representation being $\beta_{\delta }\left[n+1\right]=\beta_{\delta }\left[n\right]⊙\Delta \beta_{\delta }\left[n\right]$, $\theta_{\delta }\left[n+1\right]=\theta_{\delta }\left[n\right]⊙\Delta \theta_{\delta }\left[n\right],\delta \in \left\{rfl,rfr\right\}$, and $\omega_{u}^{n+1}=\omega_{u}^{n}⊙\Delta \omega_{u}^{n}$. The Hadamard product ⊙ and the increment Δ are fundamental to this representation.
- Rewards: The objective of this study is to minimize the transmission time of users in the colony through the implementation of an optimization strategy. To this end, negative values are employed as rewards, serving as a motivational incentive for the agents to prioritize the reduction of the transmission time. It is imperative to note that the limitations concerning the maximum speed and movement range of the UAV must be taken into consideration. Consequently, the reward function can be delineated as follows:
  (24) $$R^{n}=-D_{trans}\left[n\right]+C_{0}$$
  where indicates a constant that is imposed as a penalty when a constraint is violated.

TD3 represents an extension of the DDPG algorithm, likewise grounded in the Actor–Critic structure. Its algorithm framework encompasses an Actor policy network, $\mu_{\kappa }$, with the parameters κ, and two Critic value networks, $Q_{\zeta_{1}}$ and $Q_{\zeta_{2}}$, with the parameters $\zeta_{1}$ and $\zeta_{2}$, as well as their corresponding target networks, namely the target Actor policy network $\mu_{\kappa^{\prime }}$ and the target Critic value networks $Q_{{\zeta^{\prime }}_{1}}$ and $Q_{{\zeta^{\prime }}_{2}}$. The employment of these target networks serves to enhance the stability of the learning process by selecting the Q value that is smaller among the outputs of the two value networks during the gradient descent process. This approach is intended to alleviate the overfitting of the Q function estimate. The subsequent section will provide a detailed description of the specific implementation method of the TD3 algorithm.

At each time step, the agent calculates the output action based on the current state $s^{n}$ through the policy network $\mu_{\sigma }$:

(25) $$a^{n}=\mu_{\kappa }\left(s^{n}\right)+\rho ,\;\rho ∼N\left(0,\sigma \right)$$

where ρ is Gaussian noise. The incorporation of noise is a strategy employed to circumvent entrapment in a local optimum and enhance the efficiency of exploration. The agent uses this noisy action to interact with the environment, thereby acquiring a reward, $R^{n}$, and the next state $s^{n+1}$. It then retrieves a state transition sample, $\left(s^{n},a^{n},R^{n},s^{n+1}\right)$, and stores it in the experience replay buffer B as a training dataset for the online network.

In the event that the number of state transition samples in the experience replay buffer exceeds the preset capacity, a mini-batch containing *J* state transition samples, $\left(s^{j},a^{j},R^{j},s^{j+1}\right)$, is randomly sampled from it and used to train the online network. In contrast to the DDPG algorithm, the TD3 algorithm introduces a regularization strategy by adding truncated Gaussian noise to the actions output by the target Actor network.

(26) $$a^{\prime j+1}=\mu_{\kappa^{\prime }}\left(s^{j+1}\right)\;+\;\rho^{\prime },\;\rho^{\prime }∼\mathrm{clip}\left(N\left(0,\sigma^{2}\right),-c,c\right)$$

This Gaussian noise serves to smooth the output Q values of the two target Critic networks, thereby reducing the occurrence of overfitting, and the temporal difference (TD) error is calculated using the Bellman expectation equation based on the state–action value function:

(27) $$Q_{\mathrm{tar}}=R^{j}+\eta \min\left(Q_{{\zeta^{\prime }}_{1}}\left(s^{j+1},a^{\prime j+1}\right),Q_{{\zeta^{\prime }}_{2}}\left(s^{j+1},a^{\prime j+1}\right)\right)$$

where η denotes the discount factor, and ${\zeta_{1}}^{\prime }$ and ${\zeta_{2}}^{\prime }$ represent the parameters of the two target Critic networks. The parameters of the Critic network are updated by calculating the TD error to reduce the difference between the estimated Q value and the target Q value and by establishing two mean square error (MSE) loss functions for gradient descent:

(28) $$L\left(\zeta_{l}\right)=\frac{1}{J}\sum_{j}{\left(Q_{\zeta_{i}}\left(s^{j},a^{j}\right)-Q_{\mathrm{tar}}\right)}^{2},\;\;l=1,2$$

(29) $$\zeta_{l}\leftarrow \zeta_{l}-\lambda_{\mathrm{Critic}}\nabla_{\zeta_{l}}L\left(\zeta_{l}\right),\;l=1,2$$

where $Q_{\zeta_{l}}\left(s^{j},a^{j}\right)$ represents the estimated Q value output by the two Critic value networks and $\lambda_{Critic}$ is the learning rate.

In the context of reinforcement learning, the objective of the agent is to maximize the cumulative reward. The Q value can be utilized to measure the long-term cumulative reward of taking a specific action in the current state. During the training process, the Actor network is updated by maximizing the cumulative expected return, which is contingent upon the evaluation of the Critic network. If the Critic network demonstrates significant instability, the updating of the Actor network will be substantially impacted, resulting in system oscillation.

Therefore, the Actor network is updated with a delay, and its loss function and parameter update formula are as follows:

(30) $$J\left(\kappa \right)=-\frac{1}{J}\sum_{j}Q_{\zeta_{1}}\left(s^{j},\mu_{\kappa }\left(s^{j}\right)\right)$$

(31) $$\kappa \leftarrow \lambda_{\mathrm{Actor}}\nabla_{\kappa }J\left(\kappa \right)$$

Finally, we use a soft update strategy to synchronize the parameters of the Actor network and Critic network with the corresponding target networks:

(32) $$\zeta_{l}^{\prime }\leftarrow \tau \zeta_{l}^{\prime }+\left(1-\tau \right)\zeta_{l}^{\prime },\;l=1,2$$

(33) $$\kappa^{\prime }\leftarrow \tau \kappa +\left(1-\tau \right)\kappa^{\prime }$$

where τ is the soft update coefficient and represents the parameters of the target Actor policy network.The complete algorithm flow is shown in Algorithm 2.
**Algorithm** **2**TD3-based training algorithm 1:**Input:** $\langle {Ƶ}_{i},{Ƶ}_{k},\langle \lambda_{\mathrm{Actor}},\lambda_{\mathrm{Critic}}\rangle ,\eta ,J\rangle$ 2:**Initialize** networks, including UAV’s Actor network $\mu_{C}$, Critic network $Q_{1}$ and $Q_{2}$, and their corresponding target networks, and initialize the replay buffer B. 3:**for** episode = 1 to *M* **do** 4:     Initialize UAV’s current state $s_{t}^{i}$. 5:     **for** n=1 to $N_{do}$ **do** 6:        Select action $a^{n}=\mu_{C}\left(s^{n}\right)+\rho$ based on $s^{n}$. 7:        UAV moves according to the action $a^{n}$: to set speed, angle, STAR-RIS phase, and amplitude. 8:        Obtain $R^{n}$ and observe the $s^{n+1}$ at the next time step. 9:     Store the sample $\left(s^{n},a^{n},R^{n},s^{n+1}\right)$ in B.10:     **if** the number of samples in B > *B* **then**11:        Randomly sample a small batch from B.12:        Update the parameters $\zeta_{1}$ and $\zeta_{2}$ of Critic networks using formula (29).13:        **if** *n* mod $A_{\mathrm{update}}$ **then**14:            Update the Actor network parameters using formula (31).15:            Perform soft updates on the target network parameters using formulas (32) and (33).16:        **end if**17:     **end if**18:     **end for**19:**end for**20:**Output:** Θ, β, ω, γ

### 4.3. Computational Complexity

Combining the computational complexities of the DBSCAN clustering algorithm, the CLK path optimization algorithm, and the TD3 algorithm, the overall time complexity of the UAV path optimization algorithm can be expressed as follows: First, the DBSCAN algorithm is used for the density-based clustering of users, with a time complexity of O(NlogN), where *N* is the number of users, indicating the computational cost of the user clustering process. Next, the CLK algorithm is employed to optimize the UAV’s flight path, utilizing a local search strategy to adjust the path, with a time complexity of $O(M^{2})$, where *M* is the number of path nodes, reflecting the computational cost of the path optimization process. Finally, the TD3 algorithm is used to optimize the UAV’s trajectory and the phase and amplitude configurations of the STAR-RIS, with a time complexity of $O(T_{ep}\cdot T_{st}\cdot J\cdot \left((|S\right|+\left|A|\right)\cdot L_{B}+L_{A}\cdot L_{B}^{2}))$, where $T_{ep}$ is the number of training episodes, $T_{st}$ is the number of steps per episode, *J* is the mini-batch size, |S| and |A| are the dimensions of the state space and action space of the intelligent bodies, respectively, and $L_{A}$ and $L_{B}$ are the number of layers and the size of each layer in the network, representing the computational cost of the reinforcement learning model training process. By integrating the complexities of the DBSCAN, CLK, and TD3 algorithms, the overall time complexity of the algorithm is found to be $O(T_{ep}\cdot T_{st}\cdot J\cdot \left((|S\right|+\left|A|\right)\cdot L_{B}+L_{A}\cdot L_{B}^{2})+N\log N+M^{2})$.

## 5. Numerical Results

In this section, the proposed method is compared with several benchmark methods to evaluate its feasibility and effectiveness. The subsequent sections describe the dataset, the experimental setup, and the benchmark methods of the simulation experiment. Ultimately, the experimental results are analyzed.

### 5.1. Simulation Setting

In this study, a TD3 model was constructed based on the PyTorch 2.4.1 framework to implement the deep reinforcement learning algorithm. The server was configured with a Intel Silver (Santa Clara, CA, USA) 4210R processor (2.4 GHz), dual NVIDIA GeForce (Santa Clara, CA, USA) RTX 3080 Ti GPUs, 128 GB of memory, and a 4 TB solid-state drive.

A coverage area with a scene size of 300 m × 300 m was designed, and the position of the BS, $Z_{b}$, was set to (150,150,10), with a coverage radius of $\rho_{b}$ = 200 m. Since areas closer to the base station usually have better channel conditions, the users of the UAV service were mainly randomly distributed in the peripheral areas away from the base station and in areas outside the coverage of the base station. The task data size of the users was uniformly distributed within [1 MB, 1.5 MB]. The initial and terminal positions of the UAV flight were designated as (0,0,40) and (300, 300, 40), respectively, and the UAV moved at a constant speed of $v_{u}$ = 20 m/s. The remaining channel parameters and TD3 training parameters are enumerated in Table 2.

### 5.2. Simulation Results

**Figure 5:**
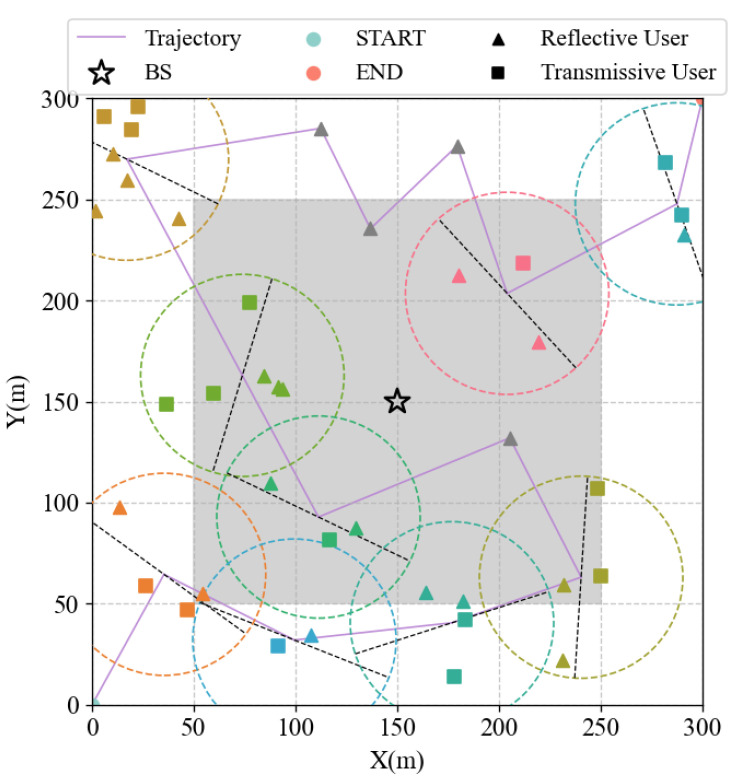
Service scenario optimization results.

As illustrated in Figure 5, the optimized user settlement, UAV trajectory, and the division of the reflection and transmission areas of each cluster (service area) are presented. Among them, the number of users, the UAV service radius, and the number of STAR-RIS units.

The utilization of the proposed algorithm enables the UAV to determine the hovering point and the optimal trajectory with precision. This is contingent upon the service radius and the user distribution. As illustrated in the figure, the UAV possesses the capacity to adaptively adjust the hovering angle in accordance with the circumstances in each settlement. This enables the UAV to cover all users in the settlement and minimize the transmission time. In particular, when a single scattered user is present, the UAV will travel to the user’s location and provide service through the reflective link.

**Figure 6:**
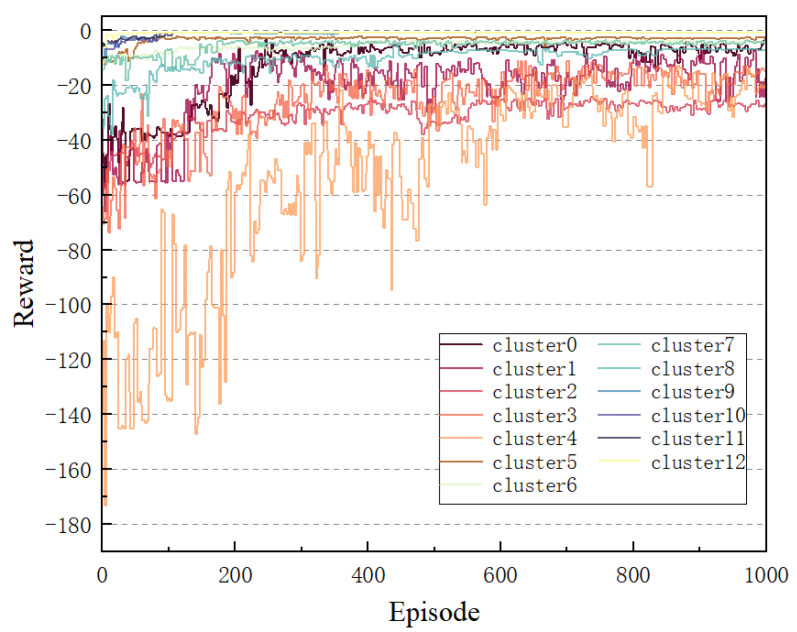
Service scenario optimization results.

As illustrated in Figure 6, the learning curve of the TD3 algorithm trained on each cluster demonstrated a clear upward trend with an increase in the number of training rounds. Despite initial fluctuations, the final reward stabilized, indicating the TD3 algorithm’s capacity to adapt effectively to a complex communication environment assisted by a STAR-RIS, thereby identifying an optimal strategy for the system. It is noteworthy that although the majority of clusters converged after approximately 200 rounds, cluster 4 exhibited a slower convergence rate and greater variability during training. This is attributable to the higher user density in cluster 4, necessitating additional iterations for the agent to converge in such a complex environment.

To assess the effectiveness of the proposed UAV-mounted STAR-RIS system in enhancing services, a comparative analysis was conducted with three baseline schemes:

- Scheme 1: Referring to [39], we considered a service enhancement scheme with a UAV equipped with an RIS and optimized it by combining maximum likelihood estimation and maximum correlation estimation. In the figure, we use “RIS” to represent this scheme.
- Scheme 2: Referring to [31], we optimized the amplitude and phase of the STAR-RIS based on the proximal policy optimization (PPO) algorithm and did not consider the hovering angle of the UAV. In the figure, we use “SRP” to represent this scheme.
- Scheme 3: Referring to [40], we optimized the amplitude and phase of the STAR-RIS without considering the hovering angle using the DDPG algorithm. In the figure, we use “SRD” to represent this scheme.

Similarly, for convenience, we use “SRT” to represent the scheme proposed in this paper.

**Figure 7:**
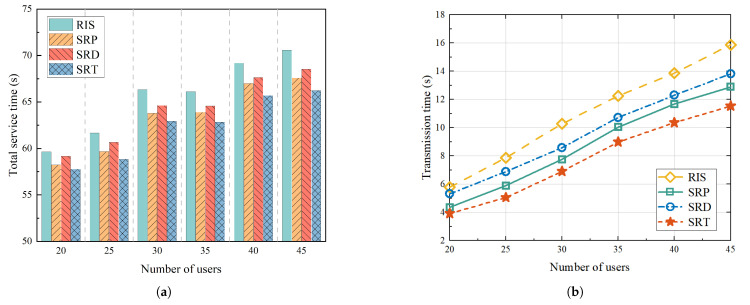
Change in time with number of users: (**a**) Shows variation in total service time for different mechanisms with different numbers of users. (**b**) Shows variation in transmission time for different mechanisms with different numbers of users.

Figure 7 illustrates the variations in the total system service time and transmission time as a function of the number of users in different mechanisms when the service radius was fixed at and the number of STAR-RIS units was fixed at 40. As the number of users increased, the total service time increased. As shown in Figure 7a, a comparison of the four mechanisms revealed that the mechanism proposed in this paper demonstrated a clear advantage when the number of users was high. Specifically, as shown in Figure 7b, when the number of users was set at 40, the transmission times of the proposed mechanism were 19.43%, 11.19%, and 15.82% lower than those of the RIS, SRP, and SRD mechanisms, respectively. This finding indicates that when the number of users in the cluster increases and inter-link interference between users increases, dynamically adjusting the STAR-RIS direction to allocate reflective and transmissive users can effectively improve system performance. Furthermore, as the number of users approached 30, a substantial increase in the total system service time was observed, primarily attributable to the increase in the flight time of the UAV surpassing the reduction in the transmission time of users. This further underscores the substantial impact of UAV flight path planning on the total system service time.

**Figure 8:**
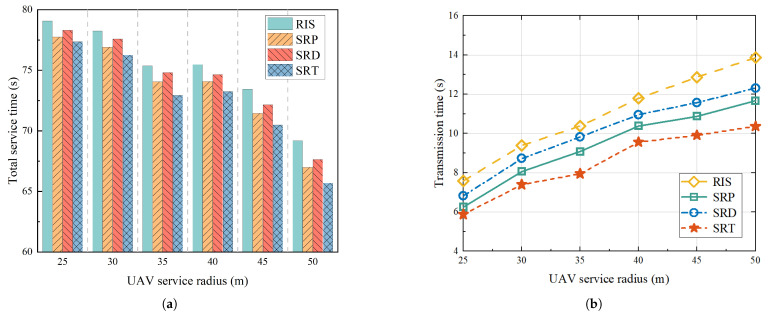
Change in time with different UAV service radius: (**a**) Shows changes in total service time for different mechanisms with different service radius. (**b**) Shows variation in transmission times for different mechanisms with different service radius.

Figure 8 illustrates the alterations in the total service time and transmission time with varying UAV service radii. As shown in Figure 8a, when the number of users was fixed at 40 and the number of STAR-RIS units was also set at 40, an increase in the service radius resulted in a gradual decrease in the UAV’s hovering points, a continuous decrease in the flight path length. As the flight time constituted a substantial fraction of the total service time, the increase in the user transmission time due to an increase in the user population within the settlement was counterbalanced by the overall reduction in the total service time. A comparison of the four mechanisms revealed that as the service radius increased, the transmission time of the proposed mechanism consistently remained low. Specifically, As shown in Figure 8b, when the UAV service radius was set to 50 m, the transmission time of the proposed mechanism was reduced by approximately 25.24% compared to the RIS mechanism, by around 11.19% compared to the SRP mechanism, and by about 15.82% compared to the SRD mechanism.

**Figure 9:**
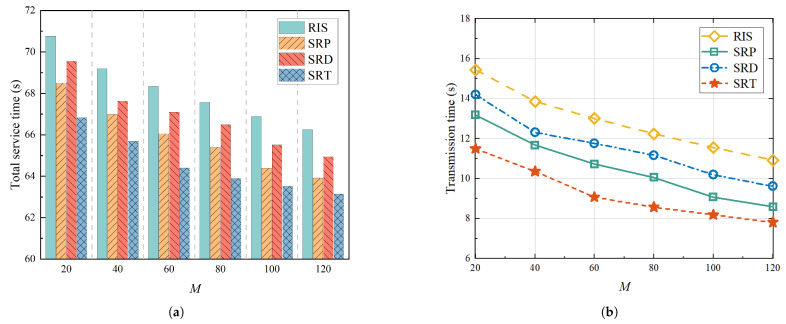
Change in time with different numbers of STAR-RIS units: (**a**) Shows variation in total service time for different mechanisms with different numbers of STAR-RIS units. (**b**) Demonstrates variation in transmission times for different mechanisms with different numbers of STAR-RIS units.

Figure 9 illustrates the system service time and transmission time with a constant service radius and 40 users, as well as a variation in the quantity of STAR-RIS units. Figure 9a demonstrates a decline in the total service time as the number of STAR-RIS units rose. Specifically, when the number of STAR-RIS units was increased from 20 to 120, the total system service time was reduced by approximately 28.0% on average. This suggests that increasing the number of units on the super-surface can effectively enhance the transmission rate of users. As shown in Figure 9b, among the four mechanisms, the RIS mechanism had the longest transmission time, which was due to the significant co-channel interference between users. The proposed mechanism in this paper consistently achieved the lowest transmission times, with reductions of 28.03%, 12.79%, and 20.15%, respectively, compared to the average transmission times of the RIS, SRP, and SRD mechanisms.

## 6. Conclusions

In this paper, we proposed a UAV service enhancement mechanism based on onboard STAR-RIS assistance, with the objective of improving the channel quality for both edge and occluded users. The proposed approach involves the joint optimization of the phase, amplitude, UAV trajectory, and hovering angle of the STAR-RIS, thereby converting the complex optimization problem into a minimization of the total service time of the UAV. Initially, the UAV service area is segmented, employing the DBSCAN algorithm, and subsequently, the optimal trajectory is derived using the CLK algorithm to minimize the flight duration. Finally, the TD3 algorithm is employed to optimize the phase, amplitude, and hovering angle of the UAV to minimize the total transmission time. The experimental results demonstrate that the proposed mechanism offers substantial advantages over traditional methodologies in terms of reducing the system service time and enhancing the user experience.

## Figures and Tables

**Table 1:** Node transfer cost matrix.

	$Z_{\mathrm{virtual}}$	$Z_{\mathrm{start}}$	$Z_{\mathrm{goal}}$	$Z_{u}^{o}$	$Z_{u}^{o^{\prime }}$
$Z_{virtual}$	*∞*	0	*∞*	*∞*	*∞*
$Z_{start}$	*∞*	*∞*	$d(e_{s,g})$	$d(e_{s,o})$	$d(e_{s,o^{\prime }})$
$Z_{goal}$	0	$d(e_{g,s})$	*∞*	$d(e_{g,o})$	$d(e_{g,o^{\prime }})$
$Z_{u}^{o}$	*∞*	*∞*	$d(e_{o,g})$	*∞*	$d(e_{o,o^{\prime }})$
$Z_{u}^{o^{\prime }}$	*∞*	*∞*	$d(e_{o^{\prime },g})$	$d(e_{o^{\prime },o})$	*∞*

**Table 2:** List of simulation parameters.

Parameter	Value
UAV altitude, $H_{u}$	40 m
Time slot length, Δt	0.5 s
Number of STAR-RIS units, *M*	40
Carrier wavelength, ν	750 MHz
Element separation gap, $\hat{d}$	ν/2
AWGN power, $\sigma^{2}$	−174 dBm/Hz
Path loss at 1 m, φ	−30 dBm
The path loss exponent, $\xi_{r,b},\xi_{k,r}$	2.2
Bandwidth, $B_{k,b}$	10 MHz
Rician factor, Υ	10 dB
User transmission power, $P_{k}$	0.1 W
Replay buffer size, B	20,000
Batch size, *J*	256
Learning rate, $\lambda_{\mathrm{Actor}},\lambda_{\mathrm{Critic}}$	0.002
Soft update factor, τ	0.05

## Data Availability

The data that support the findings of this study are available upon reasonable request from the authors.
